# RNAi‐mediated endogene silencing in strawberry fruit: detection of primary and secondary siRNAs by deep sequencing

**DOI:** 10.1111/pbi.12664

**Published:** 2017-03-04

**Authors:** Katja Härtl, Gregor Kalinowski, Thomas Hoffmann, Anja Preuss, Wilfried Schwab

**Affiliations:** ^1^ Biotechnology of Natural ProductsTechnische Universität MünchenFreisingGermany

**Keywords:** RNA interference, *Fragaria × ananassa*, transitive gene silencing, chalcone synthase, methyltransferase, RNAseq

## Abstract

RNA interference (RNAi) has been exploited as a reverse genetic tool for functional genomics in the nonmodel species strawberry (*Fragaria × ananassa*) since 2006. Here, we analysed for the first time different but overlapping nucleotide sections (>200 nt) of two endogenous genes, *FaCHS* (chalcone synthase) and *FaOMT* (*O*‐methyltransferase), as inducer sequences and a transitive vector system to compare their gene silencing efficiencies. In total, ten vectors were assembled each containing the nucleotide sequence of one fragment in sense and corresponding antisense orientation separated by an intron (inverted hairpin construct, ihp). All sequence fragments along the full lengths of both target genes resulted in a significant down‐regulation of the respective gene expression and related metabolite levels. Quantitative PCR data and successful application of a transitive vector system coinciding with a phenotypic change suggested propagation of the silencing signal. The spreading of the signal in strawberry fruit in the 3′ direction was shown for the first time by the detection of secondary small interfering RNAs (siRNAs) outside of the primary targets by deep sequencing. Down‐regulation of endogenes by the transitive method was less effective than silencing by ihp constructs probably because the numbers of primary siRNAs exceeded the quantity of secondary siRNAs by three orders of magnitude. Besides, we observed consistent hotspots of primary and secondary siRNA formation along the target sequence which fall within a distance of less than 200 nt. Thus, ihp vectors seem to be superior over the transitive vector system for functional genomics in strawberry fruit.

## Introduction

Eukaryotic organisms, including plants, animals and fungi, have developed a double‐stranded RNA (dsRNA)‐induced gene silencing mechanism to protect their cells against invading nucleic acids (Brodersen and Voinnet, 2006). In this RNA interference (RNAi) pathway, dsRNA is specifically and rapidly degraded into small interfering RNAs (siRNAs) of 21 to 25 nucleotides (nt) by RNase III‐like enzymes, known as Dicer‐like proteins (DCL; Eamens *et al*., 2008; Mlotshwa *et al*., 2008). The siRNAs are incorporated into the RNA‐induced silencing complex (RISC), which eventually leads to the degradation of any complementary single‐stranded RNA in the cytoplasm. In addition, siRNAs are recruited into RNA‐induced initiation of the transcriptional silencing complex (RITS) causing chromatin modifications (Noma *et al*., 2004). Foldback of self‐complimentary intron‐hairpin sequences (ihp), hybridization of sense and antisense sequences and the action of an RNA‐dependent RNA polymerase (RdRP) can give rise to dsRNA from endogenous, viral and transgenic RNA molecules. Studies have shown that RNAi is implicated in the suppression of transposon activity, resistance to viral infection, post‐transcriptional and post‐translational regulation of gene expression, and the epigenetic regulation of chromatin structure (Kusaba, 2004; Wang and Metzlaff, 2005).

RNAi has attracted great attention as a reverse genetic tool for studies of gene function in numerous organisms (Gilchrist and Haughn, 2010; Hoffmann *et al*., 2006; Lipardi *et al*., 2003; Zhai *et al*., 2009). The sequence specificity of RNAi‐mediated gene inactivation allows silencing of individual genes as well as multiple members of a multigene family (Miki *et al*., 2005). Transitivity, the spread of RNA silencing along primary target sequences, is an aspect of RNAi that has not been well understood (Bleys *et al*., 2006). Endogene‐derived mRNAs can become a production source of secondary siRNAs that correspond to regions of the target gene outside the original trigger (Sanders *et al*., 2002; Sijen *et al*., 2001). Secondary siRNA itself can trigger degradation of homologous transcripts through a process called transitive silencing. The production of secondary siRNAs from regions outside of the sequence initially targeted by primary siRNAs was first described in *Caenorhabditis elegans* where it proceeds over a distance of a few hundred nucleotides in the 3′–5′ direction (Sijen *et al*., 2001).

In plants, spreading of the RNAi signal has been shown to occur in both the 3′–5′ and the 5′–3′ directions along transgene RNAs in a primer‐dependent and primer‐independent manner (Bleys *et al*., 2006; Braunstein *et al*., 2002; Himber *et al*., 2003; Klahre *et al*., 2002; Kościańska *et al*., 2005; Miki *et al*., 2005; Petersen and Albrechtsen, 2005; Vaistij *et al*., 2002; Van Houdt *et al*., 2003). Silencing induced by 3′ fragments spread only for a limited distance of up to 332 nt, with a possible limit of 600 nt, while gene silencing in the 5′–3′ direction has been shown to spread over a distance of at least 1000 nt (Petersen and Albrechtsen, 2005; Vaistij *et al*., 2002). Although transitive RNA silencing of transgenes has been clearly demonstrated, many studies failed to prove transitivity along endogenous sequences. It was assumed that endogenous sequences are resistant to transitivity by some inherent properties (Vaistij *et al*., 2002; Himber *et al*., 2003; Kościańska *et al*., 2005; Miki *et al*., 2005; Petersen and Albrechtsen, 2005).

In plants, animals and fungi, RdRPs have been proposed to be a part of the RNAi silencing system (Schwach *et al*., 2005). Their likely biochemical role is the production of dsRNA, which is consistent with the *in vitro* activity of RdRPs from *Lycopersicum esculentum* (Schiebel 1998), *Neurospora crassa* (Makeyev and Bamford, 2002) and *Schizosaccharomyces pombe* (Motamedi *et al*., 2004). Models of transitive silencing suggest that RdRPs utilize siRNAs as primers and homologous transcripts as template for dsRNA synthesis (Alder *et al*., 2003; Sijen *et al*., 2001). The reaction amplifies the silencing response and mediates the production of secondary siRNAs. RdRPs add ribonucleotides to the 3′ end of a growing RNA chain, which would explain spreading of the silencing in the 3′–5′ direction along the target transcript. The 5′–3′ spreading of the silencing signal can be explained by RdRP‐mediated unprimed dsRNA synthesis at the 3′ end of target mRNAs and/or by siRNA‐primed dsRNA synthesis using an antisense transcript as template (Baulcombe, 2004; Vaistij *et al*., 2002).

In this paper, we describe the down‐regulation and the spread of silencing of two endogenous strawberry genes *Fragaria × ananassa* chalcone synthase (*FaCHS*; Lunkenbein *et al*., 2006a) and *F. × ananassa* O‐methyltransferase (*FaOMT;* Wein *et al*., 2002) by ihp constructs and transitive RNAi vectors. To systematically investigate the sequence dependency of RNAi directed to *FaOMT* and *FaCHS,* we prepared ihp constructs harbouring sequence fragments (219–303 bp) along the full lengths of both target genes. All sequence fragments resulted in a significant down‐regulation of the respective gene expression. The spreading of the silencing signal could be demonstrated by agroinfiltration of transitive RNAi vectors and was confirmed by deep sequencing of small RNAs. The data suggest that both direct ihp and transitive methods are useful tools in plant functional genomics and biotechnology, but homologous ihp sequences are superior due to their silencing efficacy, which was also demonstrated by the abundance of primary siRNAs in comparison with secondary siRNAs.

## Results

### Efficient silencing of *FaOMT* and *FaCHS* is independent of the sequence fragments used in the ihp construct

**Figure 1:**
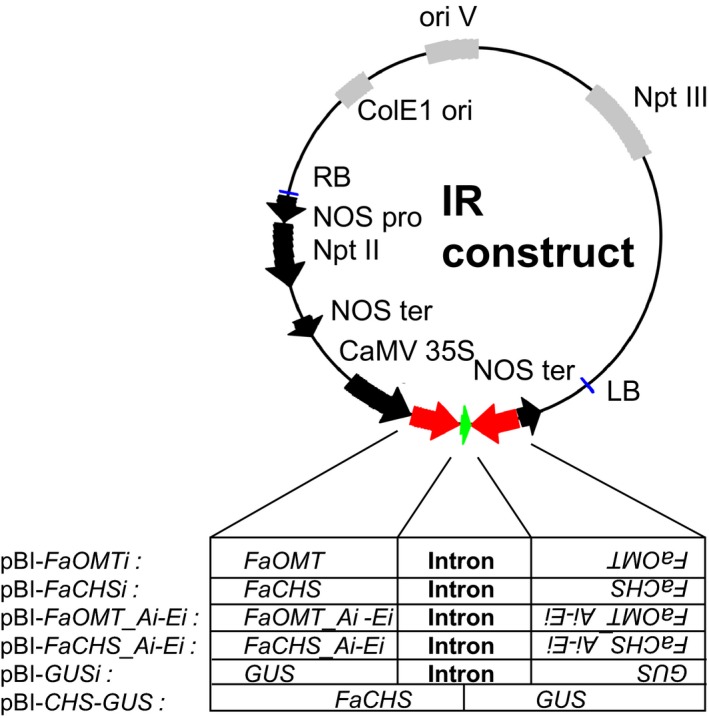
Schematic diagram of binary vectors for the determination of RNAi‐mediated *FaOMT* and *FaCHS* transcript degradation. The binary vectors shown are derivatives of pBI121. The expressed part of each plasmid relevant to this study is shown. Details about sizes and positions of the cloned fragments are summarized in Table S1.

**Figure 2:**
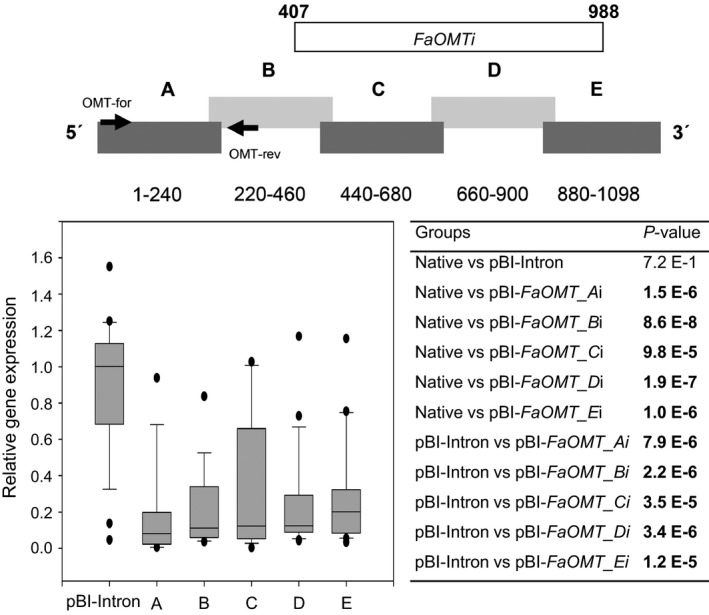
RNAi‐mediated gene silencing of *FaOMT*. Division of *FaOMT* into sections A to E with information about the nucleic acid positions, and the relative position of the PCR product obtained with primer pair OMT‐for and OMT‐rev (top). A longer fragment *FaOMTi* of 581 nt was also used (refer to Figure 7). Relative *FaOMT* gene expression in strawberry fruit infiltrated with pBI‐Intron and ihp constructs *FaOMT_Ai* to *FaOMT_Ei* (A–E) in relation to the mean expression in native untreated fruit which was set to 1 (bottom left). Number of biological replicates: native untreated fruit (*N* = 17), pBI‐Intron (*N* = 27), A (*N* = 14), B (*N* = 18), C (*N* = 18), D (*N* = 21), E (*N* = 21). The Wilcoxon–Mann–Whitney *U*‐test was used to assess intergroup significance (bottom right). Statistically significant differences (*P *< 1.0 E‐2) are shown in bold.

**Figure 3:**
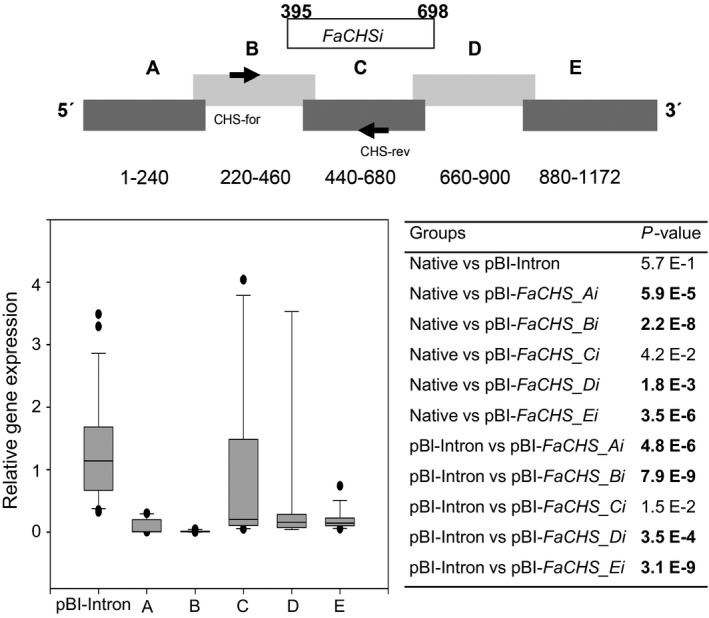
RNAi‐mediated gene silencing of *FaCHS*. Division of *FaCHS* into sections A to E with information about the nuclei acid positions and the relative position of the PCR product obtained with primer pair CHS‐for and CHS‐rev (top). Fragment *FaCHSi* was successfully used by Hoffmann *et al*. (2006). Relative *FaCHS* gene expression in strawberry fruit infiltrated with pBI‐Intron and ihp constructs *FaCHS_Ai* to *FaCHS_Ei* (A–E) in relation to the mean expression in native untreated fruit which was set to 1 (bottom left). Number of biological replicates: native untreated fruit (*N* = 23), pBI‐Intron (*N* = 26), A (*N* = 10), B (*N* = 10), C (*N* = 10), D (*N* = 9), E (*N* = 15). The Wilcoxon–Mann–Whitney *U*‐test was used to assess intergroup significance (bottom right). Statistically significant differences (*P* < 1.0 E‐2) are shown in bold.

RNA silencing with an ihp construct (pBI‐*FaCHSi*) containing a 303‐bp‐long sense and antisense sequence of *FaCHS* separated by a *Fragaria* intron sequence has been used to suppress chalcone synthase activity in strawberry (*Fragaria × ananassa*) fruit, resulting in a significantly reduced level of red fruit pigments (Hoffmann *et al*., 2006). Here, we tested the general applicability of ihp constructs to silence endogenous genes in fruits of the nonmodel strawberry plant and systematically analysed the outcome of the silencing technique, to devise a method for the application of RNAi to high‐throughput plant functional genomics in this species. In addition to *FaCHS*, the flavour‐related *FaOMT* gene (AF220491; Wein *et al*., 2002; Zorrilla‐Fontanesi *et al*., 2012) was chosen. Repression of *FaOMT* transcripts led to a near total loss of 2,5‐dimethyl‐4‐methoxy‐3(2H)‐furanone (DMMF), the product formed by the encoded enzyme from the substrate 4‐hydroxy‐2,5‐dimethyl‐3(2H)‐furanone (HDMF). In addition, lower levels of feruloyl glucose in comparison with caffeoyl glucose were observed (Lunkenbein *et al*., 2006a,b). Since it has been shown that the potency of siRNAs can vary drastically due to their thermodynamic properties, and that complex target structures may affect the activity of siRNAs (Kurreck, 2006; Overhoff *et al*., 2005; Schubert *et al*., 2005), we systematically investigated the efficiency of RNAi‐mediated gene silencing by different ihp constructs covering the complete ORFs of *FaOMT* and *FaCHS*. Both gene sequences were divided into five (A to E) overlapping fragments (Figures 1, 2, 3). These fragments were used for the assembly of the ihp constructs pBI‐*FaOMT_Ai* to *Ei* and pBI‐*FaCHS_Ai* to *Ei*. Subsequently, they were agroinfiltrated into ripening strawberry fruits, together with the control vector pBI‐Intron (Figure 1). Quantitative PCR was applied to assess the efficacy of target gene silencing (Figures 2 and 3). The relative expression levels of *FaOMT* and *FaCHS* in native versus pBI‐Intron treated fruit were statistically not significant (*P* = 7.2 E‐1 and *P* = 5.7 E‐1), indicating that injection of the control construct did not alter the expression of the target genes. However, agroinfiltration of ihp constructs resulted in a significant (*P* < 1.0 E‐2) reduction in the transcript levels of *FaOMT* (Figure 2) and *FaCHS* (Figure 3) by more than 80% (relative gene expression < 0.2) in comparison with the levels in untreated fruits, with the exception of pBI‐*FaCHS_Ci*. Although the median for pBI‐*FaCHS_Ci* is less than 0.2 (reduction by more than 80%), the *P*‐value is slightly higher than 1.0 E‐2 due to the high variance of the values. The uneven distribution of the *A. tumefaciens* strains in the tissue is an inherent problem of the agroinfiltration technique and causes high variation of the expression level. The data indicate that all five sequences comprising 240 up to 303 nt of both genes can act as efficient inducers as well as targets of post‐transcriptional silencing.

### Efficient RNA silencing manifests itself in altered metabolite levels

**Figure 4:**
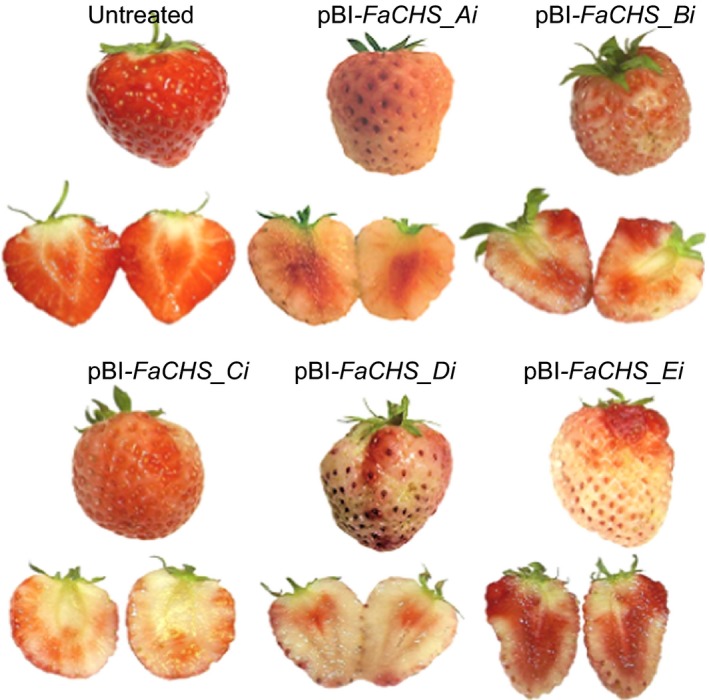
Phenotypes of untreated strawberry fruit and fruit agroinfiltrated with pBI‐*FaCHS_Ai* to pBI‐*FaCHS_Ei*.

**Figure 5:**
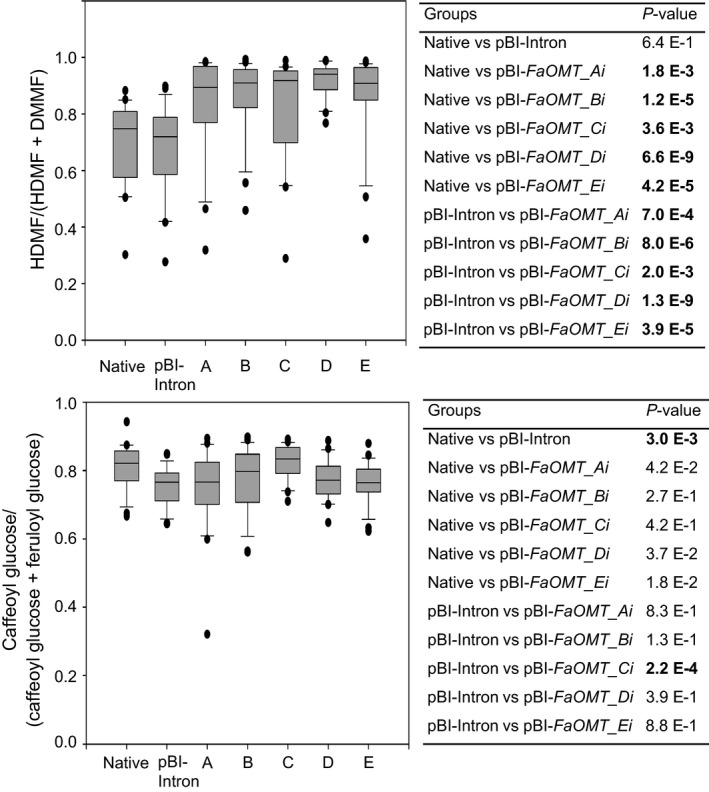
Metabolite analyses. Normalized levels of HDMF (top left) and caffeoyl glucose (bottom left) in native untreated strawberry fruit and fruit infiltrated with pBI‐Intron and ihp constructs *FaOMT_Ai* to *FaOMT_Ei* (A–E) calculated as ratio of HDMF to the total concentration of HDMF and DMMF and as ratio of caffeoyl glucose to the total concentration of caffeoyl glucose and feruloyl glucose. Number of biological replicates: native untreated fruit (*N* = 22), pBI‐Intron (*N* = 26), A (*N* = 22), B (*N* = 23), C (*N* = 21), D (*N* = 21), E (*N* = 23). The Wilcoxon–Mann–Whitney *U*‐test was used to assess intergroup significance (top right and bottom right). Statistically significant differences (*P* < 1.0 E‐2) are shown in bold.

Because *FaCHS* encodes an essential enzyme in the anthocyanin biosynthetic pathway, the reduction in *FaCHS* transcript levels results in a loss of pigmentation in infected fruits when compared to control fruits. All five ihp constructs produced a similar chimeric phenotype, indicating a significant reduction in pelargonidin 3‐*O*‐glucoside (Figure 4). This was confirmed by LC‐MS analyses (data not shown). In contrast, down‐regulation of *FaOMT* by pBI‐*FaOMT_Ai* to *Ei* did not yield an altered visible phenotype. However, LC‐MS analysis revealed differences in the ratio of HDMF to the total amount of furanones (DMMF and HDMF). Strawberries agroinfiltrated with ihp constructs targeted to *FaOMT* produced significantly lower levels (*P* < 1.0 E‐2) of the methylated product DMMF as compared with the substrate HDMF, resulting in a higher proportion of HDMF relative to the total amount of furanones (Figure 5, top). All five constructs used were similarly effective. The normalized levels of HDMF in the fruit expressing pBI‐Intron were not significantly different from the levels in untreated fruit (*P* = 6.4 E‐1).

The normalized levels of feruloyl glucose another potential product formed by FaOMT in fruit were not affected by agroinfiltration of pBI‐*FaOMT_Ai* to *Ei* when compared to the levels in untreated and pBI‐Intron infiltrated fruit (Figure 5, bottom). However, statistical analysis revealed a significant difference (*P* = 3.0 E‐3) between the normalized level in untreated and pBI‐Intron control fruit, indicating an induction of the phenylpropanoid biosynthesis by agroinfiltration. The increased formation of phenylpropanoic acids probably counteracts the metabolic effects of *FaOMT* silencing on the levels of the phenolic acids.

### Transitive silencing with transcriptional fusion of *GUS* ihp and a partial sequence of *FaCHS*

**Figure 6:**
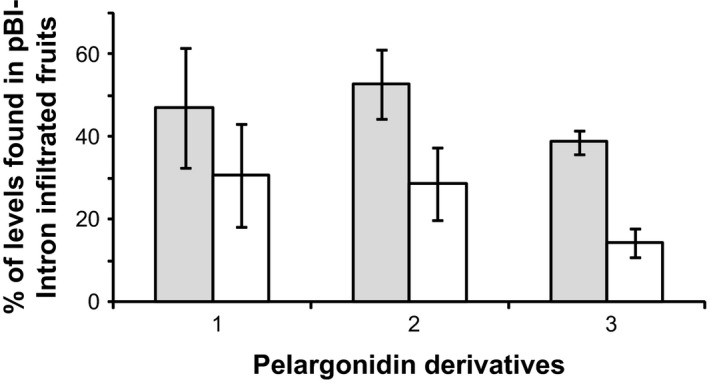
Efficacy of ihp‐PTGS versus transitive RNA silencing. Comparison of the levels of pelargonidin derivatives (1, pelargonidin 3‐*O*‐glucoside, 2, pelargonidin 3‐*O*‐glucoside‐6′‐*O*‐malonate, 3, (epi)afzelechin‐pelargonidin 3‐*O*‐glucoside) in strawberry fruit agroinfiltrated with pBI‐*FaCHSi* (white bars) and with a silencing inducer pBI‐*GUSi* in combination with pBI‐*CHS‐GUS* (grey bars) carrying one region homologous to the silencing gene (*GUS*) and another homologous to the target (*FaCHS*). Infiltration of ihp construct of *FaCHS* results in a strong down‐regulation of pelargonidin derivatives (white bars) while transitive silencing (grey bars) is less efficient.

We assembled two constructs (Figure 1) to probe whether the RNA silencing activity of a silencing inducer RNA can be transmitted to the sequence of an endogenous strawberry gene (secondary target) by fusing part of this sequence to the silencing‐inducing sequence in a single transcript (primary target; Bleys *et al*., 2006). One nucleotide sequence represented a silencing inducer and the other a primary target. A construct carrying an ihp of a partial GUS sequence (pBI‐*GUSi*) was used as source of primary siRNAs, while a fusion of the homologous GUS sequence with a *FaCHS* fragment (pBI‐*CHS‐GUS*) served as primary target (Van Houdt *et al*., 2003). T‐DNAs were introduced into ripening strawberry fruit by agroinfiltration 14 days after pollination. Strawberries harvested in the full ripe stage showed the typical chimeric phenotype of *FaCHS* deficient fruits but did not feature the extensive white sections as seen with fruit agroinfiltrated with *FaCHS* ihp constructs (data not shown). LC‐MS analysis confirmed that the levels of pelargonidin derivatives were not as strongly suppressed by transitive silencing as by ihp constructs of *FaCHS* sequences (Figure 6). In a control experiment, only the *FaCHS‐GUS* construct was agroinfiltrated but without pBI‐*GUSi*. The result clarified that the silencing effect is indeed caused by transitive silencing and not by *FaCHS‐GUS*‐induced sense cosuppression as infiltration of *FaCHS‐GUS* does not affect the pigmentation of strawberry fruit (Figure S1). It appears that transitive silencing of the endogenous *FaCHS* gene(s) by chimeric recombinant *GUS‐CHS* fusions, together with the inducer pBI‐*GUSi*, is less efficient than the down‐regulation by ihp constructs targeting directly *FaCHS* sequences.

### Detection of small RNAs by next‐generation sequencing

To test whether the reduction in endogene mRNA is caused by transitive silencing in addition to digestion with exonucleases such as XRN4, we assessed the accumulation of sequence‐specific small RNAs by next‐generation sequencing after agroinfiltration of pBI‐*FaOMT_Ci* and pBI‐*FaOMTi* into ripening strawberry fruits and focused on primary and secondary siRNAs targeting the inducer regions and the full‐length *FaOMT*.

**Figure 7:**
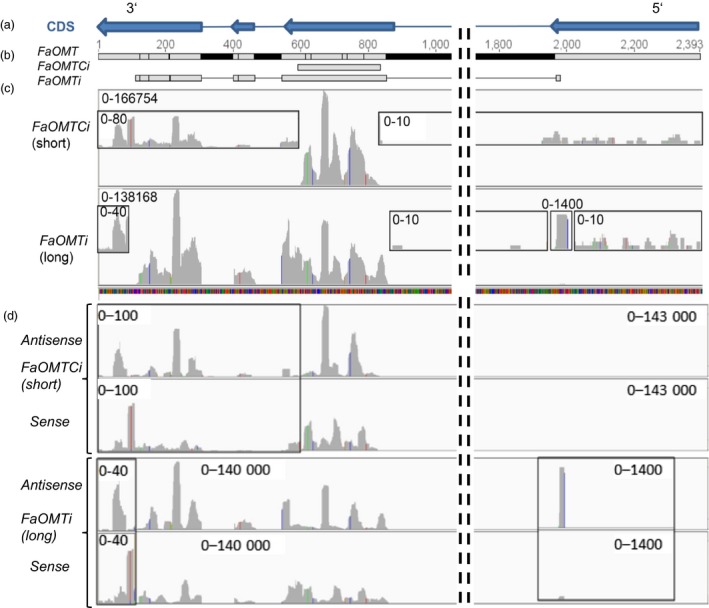
Detection of small RNAs derived from the *FaOMT* gene. Coding sequence (CDS, a). *FaOMT* gene with exons (grey)/introns (black) and number of nucleotides as well as locations of gene fragments *FaOMT_Ci* and *FaOMTi* used for the assembly of RNAi constructs (b). Abundance of siRNAs derived from different regions of *FaOMT* after agroinfiltration of the short fragment *FaOMT_Ci* and the long sequence *FaOMTi*. Insets show secondary siRNAs (c). Abundance of antisense and sense siRNAs derived from different regions of *FaOMT* after agroinfiltration of the short fragment *FaOMT_Ci* and the long sequence *FaOMTi* (d). Bar charts were computed with the Integrated Genomics Viewer (igv; Robinson *et al*., 2011). Numbers denote the range of the abundances.

We analysed the abundance of small RNAs in *FaOMT_Ci* and *FaOMTi* fruits to quantify siRNAs derived from the target sequence (primary siRNAs) and regions outside of the target (secondary siRNAs). Mapping of the quality checked sequences to the reference genome resulted in 7 245 462 (62% of total reads, *FaOMT_Ci*_short) and 11 263 154 (66% of total, *FaOMTi*_long) transcript counts, of which 9.5% (*FaOMT_ Ci* short) and 9% (*FaOMTi*_long) arise from the *FaOMT* sequence (gene 12447; accession number AF220491; Figure 7). The size distribution of total genomewide short RNAs showed the predominance of siRNAs of 21 nt and to a lesser extent of 22 nt and 24 nt, for both constructs (Figure S2a). Almost all of the siRNAs derived from *FaOMT_Ci* and *FaOMTi* were mapped to their target regions and are primary siRNAs (Figure 7c). This conclusion was supported by the dominance of short RNAs of 21 nt, 22 nt and 24 nt that mapped in the *FaOMT_Ci* and *FaOMTi* target region (Figure S2b). However, low‐abundant short RNAs were also visible upstream (5′) of both, the *FaOMT_Ci* and *FaOMTi* target sequences, and in particular downstream (3′) of both inducer sequences (Figure 7c insets), confirming the formation of secondary siRNAs. They almost exclusively consisted of short RNAs of 21 nt and 22 nt (Figure S2c).

There is uneven, nonrandom distribution of siRNAs within the target sequences suggesting the presence of hotspots for siRNA formation. Interestingly, the positions of these hotspots differed between the *sense* and *antisense* strands but not between primary and secondary siRNAs (Figure 7d). The similarity of the abundance profiles but not the absolute counts of the secondary siRNAs produced by *FaOMT_Ci* and the primary siRNAs evoked by *FaOMTi* indicates that related enzymes are involved in the cleavage of the precursor RNAs. The size distribution of siRNAs mapped in the inducer regions *FaOMT_Ci* and *FaOMTi* revealed, almost exclusively, siRNAs of 21, 22 and 24 nt (Figure S2b).

## Discussion

We have exploited RNAi as a reverse genetic tool to develop a rapid system to perform functional genomics in strawberry fruit (F.* × ananassa*) (Griesser *et al*., 2008; Hoffmann *et al*., 2006; Matthew, 2004; Song *et al*., 2015). To systematically investigate the silencing efficacy of ihp constructs that carry different gene sequence segments, we divided the ORFs of *FaCHS* and *FaOMT* into five fragments (240–293 nt). In total, ten vectors were assembled each containing the nucleotide sequence of one fragment in sense and corresponding antisense orientation separated by an intron obtained from a *F. × ananassa* quinone oxidoreductase gene. Bioinformatic analysis of the modelled mRNA structures suggested different silencing efficiencies of the sequences A to E due to strongly varying theoretical accessibilities (ranging from 0.48 to 1.90 for *FaOMT* fragments and 0.05 to 1.88 for *FaCHS* fragments) of local target sites as has been reported by Schubert *et al*., 2005;. Accessible local target sites are defined as mRNA regions with more than 10 unpaired nt (Overhoff *et al*., 2005). However, the efficiencies of the applied constructs to silence the endogenous *FaCHS* and *FaOMT* genes did not show significant differences. Thus, it appears that additional factors govern the efficiency of the post‐transcriptional silencing process in plant cells. Until now, all characterized sRNAs get incorporated into ARGONAUTE (AGO) proteins to contribute to RISC formation (Fang and Qi, 2016). Recently, it was suggested that subcellular compartmentalization of AGO‐miRNA‐RISC complexes and sequestration of miRNAs and siRNAs could be additional factors determining the silencing efficiency (Fang and Qi, 2016; Wu *et al*., 2015).

Although our results did not show any significant difference in the silencing efficiency of the various constructs, the uneven distribution of primary siRNAs along the target sequence (Figure 7), that is the detection of siRNA hotspots and low‐abundant siRNAs, suggests that the use of shorter inducer sequence would eventually lead to unequal silencing efficacies. As the distance between siRNA hotspots is less than 200 nt inducer, sequences of more than 200 nt in length are sufficient to initiate the stable down‐regulation of endogenous genes.

Quantitative PCR analyses with primer pairs specific to the 5′ regions of the target RNAs (located in *FaOMT* fragment A to B and *FaCHS* fragment B to C) revealed substantial degradation of primer target sites after agroinfiltration of all ihp RNA sequences used (Figures 2 and 3). Degradation of mRNA outside the primary RNAi target sites might be explained by digestion of the aberrant mRNA with exonucleases such as XRN4 (Vazquez and Hohn, 2013). Alternatively, this observation might provide a hint of the spreading of the RNAi signal from the 3′ to the 5′ region over a distance of up to 1000 nt and in the reverse direction of up to 500 nt. Spreading over a distance of at least 1000 nt from the 5′ end to the 3′ end has been shown by virus‐induced gene silencing (VIGS), while 3′–5′ spreading extended at least through 332 nt, with a possible limit of 600 nt (Petersen and Albrechtsen, 2005; Vaistij *et al*., 2002). Thus, agroinfiltration of ihp constructs appears to be equally effective in spreading of the RNAi signal as VIGS.

It has been proposed that the interaction of sense transcripts with the antisense siRNAs might change the structure of the RNA or of a ribonucleoprotein complex and thereby allow RdRP to access the 3′ end of the target RNA. Synthesis of dsRNA from the 3′ end would result in siRNA production corresponding to the entire transcript sequence and could explain RdRP‐mediated spreading in a primer‐independent way (Vaistij *et al*., 2002).

There are only a few reports showing the spread of RNAi along endogenous genes as we have shown by agroinfiltration of transitive vectors into strawberry fruit (Figure 6). By contrast, transitivity has frequently been observed along reporter transgenes (Sanders *et al*., 2002). Since most studies failed to demonstrate spreading along endogenous plant genes, it has been concluded that transgenes, and not endogenous genes, are good templates for generation of secondary siRNAs. Reasons for the plant RdRP's apparent preference for the transgene transcripts as a template in the transitivity remained unclear (Miki *et al*., 2005; Petersen and Albrechtsen, 2005; Shimamura *et al*., 2007; Vaistij *et al*., 2002). Transgenes and RNAi probes are mostly expressed under the control of strong promoters (e.g. CaMV 35S). Thus, the primary silencing inducer targeting the transgene gives rise to a large number of primary siRNAs that eventually exceed a critical threshold level, which may lead to the activation of RdRP, and finally to efficient transitive silencing. Consistent with this hypothesis, it has been shown that a hairpin construct controlled by the strong 35S promoter induced a stronger silencing phenotype than the same construct controlled by the weak nopaline synthase promoter (Chuang and Meyerowitz, 2000). In addition, the efficiency of transitive silencing of endogens depends on the degree of sequence homology to the primary target (Bleys *et al*., 2006). However, strong silencing of the target RNA would mean that the template RNA for RdRP is scarce, which would also reduce the efficiency of spreading (Vaistij *et al*., 2002). Taken together, both the level of siRNAs and the target transcript might determine the onset of transitivity. Transcript levels of numerous endogenous genes are less abundant and therefore less prone to transitive silencing (Vaistij *et al*., 2002).

Attempts at explaining the absence of RNA silencing of the phytoene desaturase (*PDS*) endogenous gene have evoked the characteristics of endogenous RNAs that inhibit RdRP or prevent interactions with siRNAs (Vaistij *et al*., 2002). However, in the meantime, *PDS* alleles have been successfully down‐regulated by a hairpin construct in sugarcane and tobacco, which invalidates the former hypothesis (Osabe *et al*., 2009; Wang *et al*., 2010). Thus, the assumption that transgenes are better templates for production of secondary siRNAs is no longer considered a likely explanation for the inability to detect transitivity in certain studies. Our results support the hypothesis that there is no principle distinction between transgenes and endogenous genes, except their promoters with respect to their active recruitment in RNA silencing including spreading of the signal (Sanders *et al*., 2002).

In *Arabidopsis*, it has been shown that both transgenes and endogenes can be silenced by secondary transitive signals (Bleys *et al*., 2006). Since insertion of additional nt sequences between the region targeted by the silencing inducer and the upstream region homologous to a transgenic target (*GUS*) led to a delay of the onset of transitive silencing, it has been suggested that transitivity requires time to accumulate a certain steady state level of secondary siRNAs that results in a corresponding maximum plateau level of silencing. Thus, ihp constructs driven by a strong promoter will reach the level earlier.

RNA silencing was discovered in plants as a mechanism whereby invading nucleic acids such as viruses are suppressed. In this context, it appears logical that transgenes that are expressed under the control of viral promoters (e.g. CaMV 35S) are efficiently silenced by transitive RNAi, while endogenous transcripts seem to be protected (Bleys *et al*., 2006). This observation supports the proposed biological function of this process as a natural antiviral response. Spreading of the RNAi signal by transitive silencing even intensifies the efficacy of viral gene suppression. However, it could also lead to uncontrollable degradation of plant genes that show partial homology to the inducer sequence. In strawberry, the introduction of the *Vitis vinifera* stilbene synthase gene under the control of the 35S promoter inadvertently caused the down‐regulation of the endogenous chalcone synthase gene transcripts, probably due to transitive silencing (Hanhineva *et al*., 2009).

To detect primary and secondary siRNAs and to clearly prove the spreading of the RNAi signal, deep sequencing of small RNAs in *FaOMT_Ci* and *FaOMTi* fruits was performed for the first time in strawberry fruit and revealed 21, 22 and 24 nt small RNAs (Figure 7d) as the most dominant sizes, consistent with the siRNA size distribution observed in angiosperms (Chávez Montes *et al*., 2014). The high percentage of 21nt RNAs indicated that they were predominantly produced by the activity of DCL4‐like nucleases (Fusaro *et al*., 2006). DCL2 and DCL3 can substitute DCL4, producing 22nt and 24nt RNAs (Ghildiyal and Zamore, 2009; Small, 2007). In plants, AGO proteins sort miRNAs and siRNAs based on size and the identity of the 5′ nucleotide. 21‐mers typically associate with AGO1 and guide mRNA cleavage and subsequent secondary siRNA production, whereas 24‐mers associate with AGO4 and 6 promoting the formation of repressive chromatin (Ghildiyal and Zamore, 2009). Furthermore, it has been shown that also 22nt miRNAs can trigger siRNA production, but apparently asymmetrically positioned bulged bases in the miRNA:miRNA* duplex within the AGO proteins are sufficient to trigger transitivity (Manavella *et al*., 2012). When only small RNAs were considered that mapped into their target sequence (*FaOMT_Ci* and *FaOMTi*), a marked reduction of the proportion of long RNAs (>25 nt) was observed but a substantial number of short RNAs (<21 nt) were still counted (Figure S2). Interestingly, 9.5% and 9% of the siRNAs isolated from *FaOMT_Ci* and *FaOMTi* fruits, respectively, targeted the *FaOMT* sequence. Thus, the RNAi machinery was efficiently recruited by the ihp vectors. The proportion of long RNAs (>21 nt) was increased in *FaOMTi* fruits in comparison with *FaOMT_Ci* samples, whereas the percentage of short RNAs (<21 nt) was higher in *FaOMT_Ci* fruit. The reason for this observation is unclear, but the very short RNAs might be secondary degradation products of siRNAs.

Clustering of siRNAs along the inducer sequences was observed, indicating that siRNAs are apparently nonrandomly distributed (Figure 7). Similar asymmetry in siRNA distribution has been observed in studies on the RNAi‐mediated silencing of the GFP gene (Llave *et al*., 2002) and plant virus‐induced RNA silencing (Molnár *et al*., 2005). One explanation is that loading of siRNAs into AGO proteins, which are part of the RISC complex, is specified by the 5′ terminal nucleotide as most 21 nt siRNAs with 5′ terminal U are predominantly associated with AGO (Mi *et al*., 2008). Secondly, the structure of the dsRNA might determine the specificity and efficiency of DCL activity (Vermeulen *et al*., 2005). Besides, it was shown that the position of transacting siRNA‐generating loci (*TAS* genes) can restrict siRNA production to certain regions (Rajeswaran *et al*., 2012). Also the distinctive distribution pattern of mapped short sequences in *FaOMT_Ci* and *FaOMTi* infiltrated fruits indicates certain hotspots of siRNA production (Figure 7c and d).

Primary siRNAs were identified by mapping to the inducer sequence, whereas secondary siRNAs are produced from regions outside of the sequence initially targeted by primary siRNAs. Low‐abundant secondary siRNAs were detected upstream and in particular downstream of the inducer sequences (Figure 7c insets) indicating that formation by primer‐independent synthesis by RdRP strongly prevails. The low numbers of small RNAs upstream of the inducer sequence make transitive silencing by siRNA‐primed cRNA synthesis very unlikely. Similarly, silencing induced by a central ß‐glucuronidase (GUS) gene fragment in *Nicotiana benthamiana* carrying a GUS transgene spread only into downstream regions (Petersen and Albrechtsen, 2005), and secondary siRNAs originated preferentially from the 3′ region of the inducer region in tobacco (Shimamura *et al*., 2007). However, in *Caenorhabditis elegans,* it has been shown that secondary siRNA has a 5′ triphosphate, precluding cloning methods that rely on a single 5′ phosphate (Miska and Ahringer, 2007). Thus, this species might be underrepresented in the large‐scale sequencing studies. Di‐ and triphosphate groups at the 5′ termini of secondary siRNAs also indicate that they are likely to be primary, unprimed RdRP products (Carthew and Sontheimer, 2009).

The strong but similar sense/antisense bias of primary and secondary siRNAs (Figure 7d) suggests a related formation pathway. It is conceivable that secondary siRNAs are also formed by DCL4 3′ of the primary targeted *FaOMT*, as mRNA cleavage, rather than priming of RdRP by primary siRNAs, was proposed as the signal for siRNA amplification (Ghildiyal and Zamore, 2009).

Overall, in our study, primary siRNAs were three orders of magnitude more abundant than secondary siRNAs (Figure 7) confirming the observation that ihp constructs, compared with the transitive construct, generated higher frequencies (data not shown) and efficacies of loss‐of‐function phenotypes (Filichkin *et al*., 2007; Figure 6).

Our results demonstrate that endogenous genes can be efficiently down‐regulated in strawberry fruit by RNAi‐mediated silencing after agroinfiltration of ihp constructs carrying sequences of 200 up 300 nt homologous to an endogene within 14 days. Transitive vectors are less effective probably due to lower levels of secondary siRNAs targeting the gene of interest. Since the silencing efficiency is independent of the calculated theoretical accessibilities of the nucleotide sequences used, and thus independent of the gene fragment, the method represents a versatile tool to perform functional genomics research in strawberry.

## Materials and methods

### Constructs

The pBI‐Intron control construct was assembled as described by Hoffmann *et al*. (2006). pBI‐Intron contained *GUS* separated by the first intron of the *F. × ananassa* quinone oxidoreductase gene (AY158836, nucleotides 4107–4561). The assay constructs were made by using pBI121 (Jefferson, 1987). The second intron of the *F. × ananassa* quinone oxidoreductase gene (AY158836, nucleotides 4886–4993) was PCR‐amplified from strawberry *F. × ananassa* cv. Elsanta genomic DNA and cloned into BamHI–Ecl136II cut pBI121 to replace *GUS*. For cloning of the *FaCHS* and *FaOMT* silencing constructs, the genes were divided into five parts, respectively, that overlapped each other to ensure complete coverage of the full sequences (Figures 2 and 3). These parts were PCR‐amplified by primers introducing restriction sites and overhanging nucleotides. After restriction digestion, the pieces were inserted into the 5′ and 3′ arms of the intron in sense and antisense direction allowing the formation of self‐complementary ihp structures. The resulting plasmids were named pBI‐*FaCHS_Ai* to pBI‐*FaCHS_Ei* and pBI‐*FaOMT_Ai* to pBI‐*FaOMT_Ei*. To generate pBI‐*GUSi,* a 414‐bp fragment of *GUS* (AF485783) was inserted into the 5′ and 3′ arms of the intron. For pBI‐*CHS*‐*GUS* construction, a 244‐bp *CHS* fragment was PCR‐amplified and cloned into SnaBI‐Ecl136II‐cut pBI121 (Jefferson, 1987). All primer sequences including restriction sites, amplicon sizes and amplicon positions are summarized in Table S1.

### Plant material

The octoploid strawberry *F. × ananassa* cv. Elsanta (Kraege Beerenobst, Telgte, Germany) was purchased as frigo plants (Kraege, Telgte, Germany) and used for transient gene experiments (Hoffmann *et al*., 2006). Standard growing conditions were maintained at 25 °C and a 16‐h photoperiod under 120 μmol/m^2^/s irradiance provided by Osram Fluora lamps (München, Germany). For genetic and molecular analysis, fruits were injected 14 days after pollination and harvested 3–24 days after injection. Fruits harvested 28 days after pollination were used as controls.

### Agroinfiltration

Infiltration of Agrobacterium AGL0 (Lazo *et al*., 1991) containing the different constructs was performed according to Hoffmann *et al*. (2006) and Spolaore *et al*. (2001).

### qPCR

Total RNA was extracted from mature fruit using the cetyltrimethylammonium bromide (CTAB) extraction procedure (Asif *et al*., 2000; Liao *et al*., 2004). RNA samples were treated with RNase‐free DNase I (Fermentas, St. Leon‐Rot, Germany) for 1 h at 37 °C. First‐strand cDNA synthesis was performed in duplicate in a 20 μL reaction volume, with 1 μg of total RNA as the template, random primer (random hexamer, 100 pmol) and M‐MLV reverse transcriptase (200 U, Invitrogen, Karlsruhe, Germany) according to the manufacturer's instructions. Real‐time PCR was performed with 2 μL of cDNA on a StepOnePlus System (Applied Biosystems, Foster City, CA) using SYBR Green PCR Master MIX (Applied Biosystems). To monitor dsDNA synthesis, data were analysed with ABI StepOne Software v2.0. Relative quantification of the *FaCHS* (AI795154) and *FaOMT* (AF220491) transcripts was performed using the *Actin* gene (AB116565) from *F. × ananassa* as a reference (Almeida *et al*., 2007). Quantitative PCR primers can be found in Table S1, with supplemental information about amplicon lengths and positions. All reactions were run two times with two sets of cDNAs. The specificity of the PCR amplification was checked with a melting curve analysis following the final step of the PCR. For each sample, threshold cycles (Ct, cycle at which the increase of fluorescence exceeded the threshold setting) were determined. Relative gene expression data were normalized to the mean expression of *FaOMT* (*n* = 17) and *FaCHS* (*n* = 23) in native, untreated fruits (set to 1). The relative expression ratio was calculated according to Pfaffl (2001).

### Metabolite analysis

Identification and quantification of strawberry fruit metabolites were performed by liquid chromatography‐UV‐electrospray ionization‐mass spectrometry (LC‐MS) as described by Hoffmann *et al*. (2006) and Griesser *et al*. (2008). Box plots of signal intensities were generated by Sigma Plot (SPSS, Chicago, IL), and statistical significance levels were calculated using the Wilcoxon–Mann–Whitney *U*‐test (Hart, 2001).

### Bioinformatic analysis and processing of transcriptomic data

PBi‐*FaOMTi* and pBi‐*FaOMT_Ci* were agroinfiltrated into ripening strawberry fruit according to Hoffmann *et al*. (2006). Total RNA was extracted from mature fruit using the CTAB extraction procedure (Asif *et al*., 2000; Liao *et al*., 2004). The RNA samples were treated with RNase‐free DNase I (Fermentas, St. Leon‐Rot, Germany) for 1 h at 37 °C and sent to Eurofins Genomics (Ebersberg, Germany), where library preparation and RNA sequencing were carried out. The library was prepared with the TruSeq(TM) SBS v5 kit (Illumina Inc.). Final cDNA libraries were purified and size‐selected by capillary electrophoresis (<51 nt). Sequencing was performed on an Illumina HiSeq 2000 platform after a PhiX library (Illumina Inc.) was spiked in the channel to estimate the sequencing quality. The following software packages were used for base calling: HiSeq control software v. 1.4.8, RTA 1.12.4.2, CASAVA 1.7.0 and OLB‐1.9.0 (all Illumina Inc.). Prior to read sorting, the 3′ adaptor sequence was trimmed from the raw reads using cutadapt 0.9.3 (Martin, 2011). Sequencing (<50 nt) yielded 14 288 304 reads (OMT_short) and 21 722 563 reads (OMT_long), respectively.

Subsequent data processing was performed on the Galaxy server (Blankenberg *et al*., 2010b; Giardine *et al*., 2005; Goecks *et al*., 2010). The application programming interface (API) was used to set up pipeline analyses (Sloggett *et al*., 2013), and the Galaxy Data Manager was employed for handling built‐in reference data (Blankenberg *et al*., 2014a). Tools were installed and maintained via Galaxy ToolShed (Blankenberg *et al*., 2014b). Before mapping, reads were filtered according to their quality score and length (15–50 nt; min. quality 20) with the fastq_filter v. 1.0.0 tool implemented in Galaxy (Blankenberg *et al*., 2010a). Overall read quality was tracked with the FastQC software by S. Andrews (http://www.bioinformatics.babraham.ac.uk/projects/fastqc/).

After quality clipping, 11 693 897 (81.84% of total reads, OMT_short) and 17 084 937 (78.65%, of total reads OMT_long) reads were subjected to the mapping against the *F. vesca* reference genome (version 2.0.a1: downloaded from Genome Database for Rosaceae GDR www.rosaceae.org; Tennessen *et al*., 2014). Mapping was done by TopHat read aligner v 2.0.14 (Kim *et al*., 2013) for single‐end data in default settings. Aligned reads were counted from resulting bam files by HTSeq‐count v. 0.6.0 (Anders *et al*., 2015) in “Union” mode for stranded reads with a minimum alignment quality of 10. The gene prediction input file was also downloaded from GDR (Tennessen *et al*., 2014). The coverage of small RNAs was visualized with the Integrated Genomics Viewer (igv, Robinson *et al*., 2011).

### Modelling of mRNA structures

Modelling of the *FaCHS_Ai* to *Ei* and *FaOMT_Ai* to *Ei* mRNA structures and calculation of the theoretical accessibilities was carried out online via the *Mfold* web server for nucleic acid folding and hybridization prediction (http://unafold.rna.albany.edu/?q=mfold/RNA-Folding-Form) in default parameters as published by Zuker (2003).

## Conflict of interest

The author(s) declare that they have no competing interests.

## Funding

This work was supported by the DFG (grant no. SCHW634/10‐2, SCHW634/24‐1) and BMBF (grant no. PLANT‐KBBE, FraGENOMICS, 0315463).

## Supporting information


**Figure S1** Control experiments. Infiltration of pBI‐*FaCHS‐GUS* to verify that the chimeric effect is caused by transitive silencing, and not by *FaCHS‐GUS*‐induced sensed co‐suppression (right). Successful down‐regulation of *CHS* as demonstrated by the partial loss of pigmentation was shown by agroinfiltration of a pBI‐*FaCHSi* construct (left).
**Figure S2** Size distribution of total genome‐wide short RNAs (a), short RNAs mapped in the *FaOMT_C*i and *FaOMTi* target region (b), and short RNAs mapped outside of the target regions but within the full length *FaOMT* sequence (c). Relative proportions of short RNA sequences are shown as percentages for each size category.
**Table S1** Primer sequences used for the cloning and quantification of the different *FaCHS* and *FaOMT* fragments, including the length of the amplicons in nucleotides (nt), and their positions on the respective genes. Restriction sites are denoted in bold letters.
