# Supplementary material for: RNAi‐mediated endogene silencing in strawberry fruit: detection of primary and secondary siRNAs by deep sequencing
Source: Plant Biotechnol J. 2017 Mar 4;15(5):658–68. doi: 10.1111/pbi.12664 (PMC5398998; doi:10.1111/pbi.12664)
Supplement: Supplementary file 1 — Figure S1 Control experiments. Infiltration of pBI‐FaCHS‐GUS to verify that the chimeric effect is caused by transitive silencing, and not by FaCHS‐GUS‐induced sensed co‐suppression (right). Successful down‐regulation of CHS as demonstrated by the partial loss of pigmentation was shown by agroinfiltration of a pBI‐FaCHSi construct (left). Figure S2 Size distribution of total genome‐wide short RNAs (a), short RNAs mapped in the FaOMT_Ci and FaOMTi target region (b), and short RNAs mapped outside of the target regions but within the full length FaOMT sequence (c). Relative proportions of short RNA sequences are shown as percentages for each size category. Table S1 Primer sequences used for the cloning and quantification of the different FaCHS and FaOMT fragments, including the length of the amplicons in nucleotides (nt), and their positions on the respective genes. Restriction sites are denoted in bold letters. [file PBI-15-658-s001.pptx]

## Slide 1
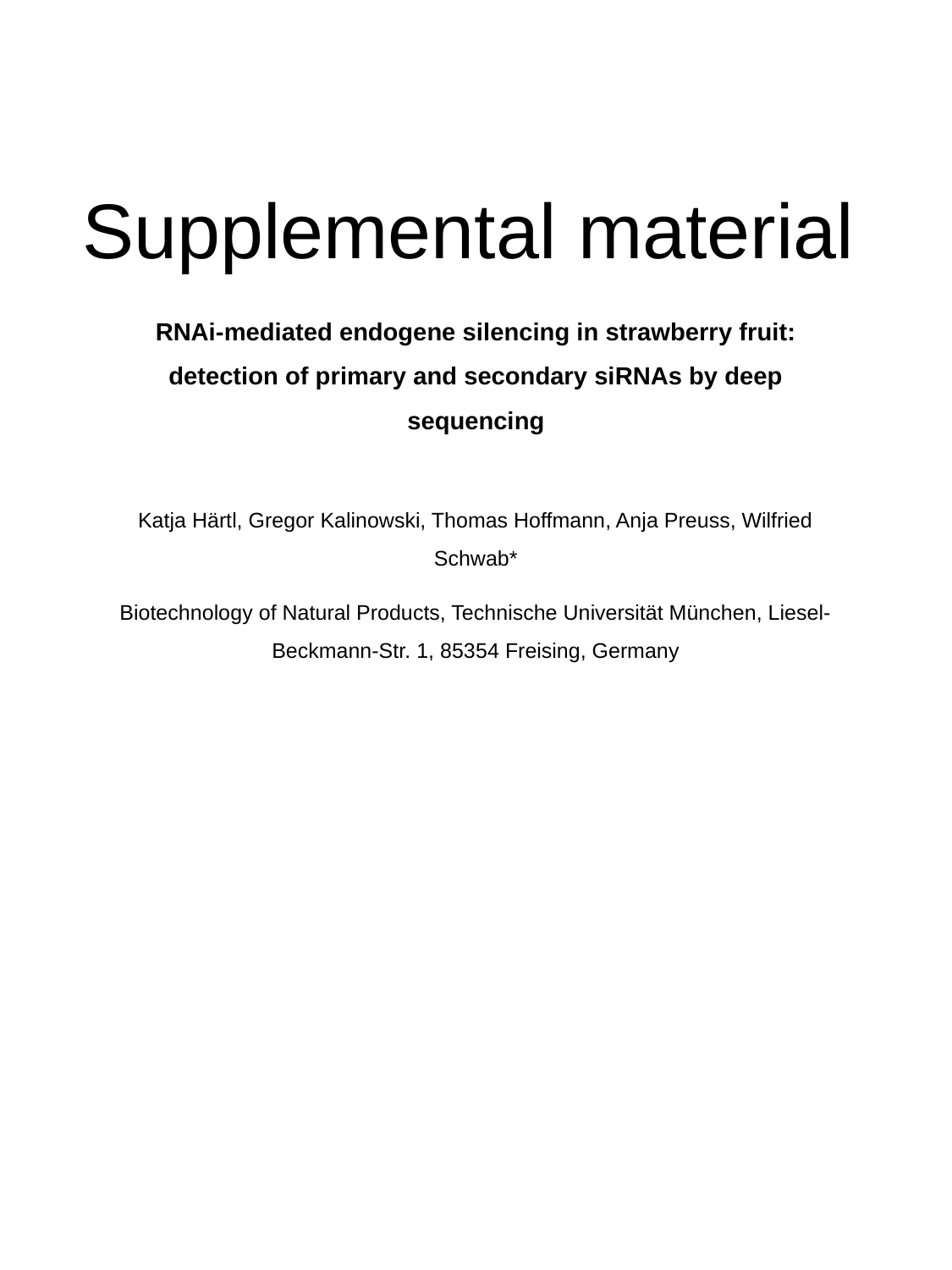

Supplemental material
RNAi-mediated endogene silencing in strawberry fruit: detection of primary and secondary siRNAs by deep sequencing
Katja Härtl, Gregor Kalinowski, Thomas Hoffmann, Anja Preuss, Wilfried Schwab*
Biotechnology of Natural Products, Technische Universität München, Liesel-Beckmann-Str. 1, 85354 Freising, Germany

## Slide 2
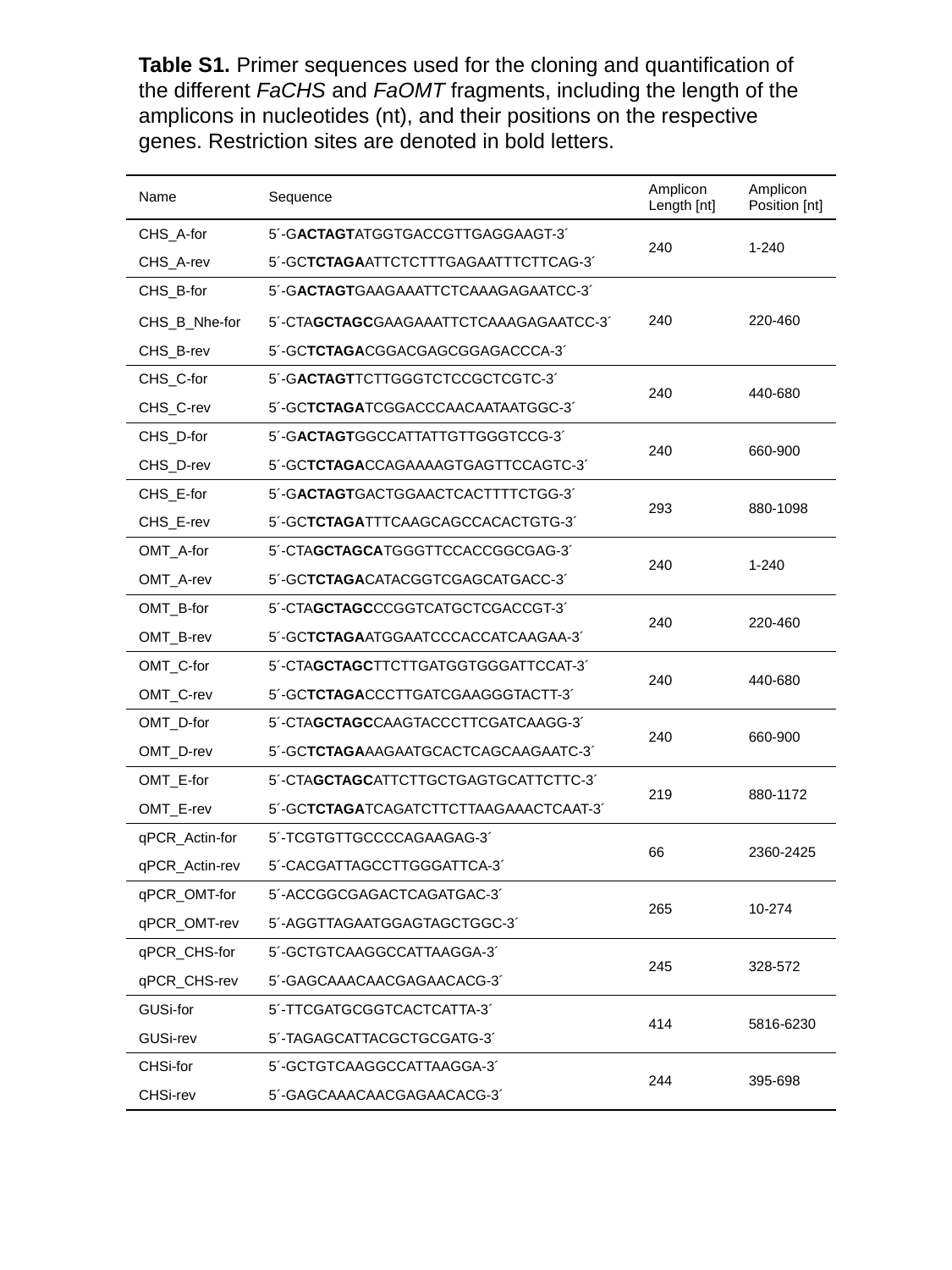

Table S1. Primer sequences used for the cloning and quantification of the different FaCHS and FaOMT fragments, including the length of the amplicons in nucleotides (nt), and their positions on the respective genes. Restriction sites are denoted in bold letters.
| Name | Sequence | Amplicon Length [nt] | Amplicon Position [nt] |
| --- | --- | --- | --- |
| CHS\_A-for | 5´-GACTAGTATGGTGACCGTTGAGGAAGT-3´ | 240 | 1-240 |
| CHS\_A-rev | 5´-GCTCTAGAATTCTCTTTGAGAATTTCTTCAG-3´ | | |
| CHS\_B-for | 5´-GACTAGTGAAGAAATTCTCAAAGAGAATCC-3´ | 240 | 220-460 |
| CHS\_B\_Nhe-for | 5´-CTAGCTAGCGAAGAAATTCTCAAAGAGAATCC-3´ | | |
| CHS\_B-rev | 5´-GCTCTAGACGGACGAGCGGAGACCCA-3´ | | |
| CHS\_C-for | 5´-GACTAGTTCTTGGGTCTCCGCTCGTC-3´ | 240 | 440-680 |
| CHS\_C-rev | 5´-GCTCTAGATCGGACCCAACAATAATGGC-3´ | | |
| CHS\_D-for | 5´-GACTAGTGGCCATTATTGTTGGGTCCG-3´ | 240 | 660-900 |
| CHS\_D-rev | 5´-GCTCTAGACCAGAAAAGTGAGTTCCAGTC-3´ | | |
| CHS\_E-for | 5´-GACTAGTGACTGGAACTCACTTTTCTGG-3´ | 293 | 880-1098 |
| CHS\_E-rev | 5´-GCTCTAGATTTCAAGCAGCCACACTGTG-3´ | | |
| OMT\_A-for | 5´-CTAGCTAGCATGGGTTCCACCGGCGAG-3´ | 240 | 1-240 |
| OMT\_A-rev | 5´-GCTCTAGACATACGGTCGAGCATGACC-3´ | | |
| OMT\_B-for | 5´-CTAGCTAGCCCGGTCATGCTCGACCGT-3´ | 240 | 220-460 |
| OMT\_B-rev | 5´-GCTCTAGAATGGAATCCCACCATCAAGAA-3´ | | |
| OMT\_C-for | 5´-CTAGCTAGCTTCTTGATGGTGGGATTCCAT-3´ | 240 | 440-680 |
| OMT\_C-rev | 5´-GCTCTAGACCCTTGATCGAAGGGTACTT-3´ | | |
| OMT\_D-for | 5´-CTAGCTAGCCAAGTACCCTTCGATCAAGG-3´ | 240 | 660-900 |
| OMT\_D-rev | 5´-GCTCTAGAAAGAATGCACTCAGCAAGAATC-3´ | | |
| OMT\_E-for | 5´-CTAGCTAGCATTCTTGCTGAGTGCATTCTTC-3´ | 219 | 880-1172 |
| OMT\_E-rev | 5´-GCTCTAGATCAGATCTTCTTAAGAAACTCAAT-3´ | | |
| qPCR\_Actin-for | 5´-TCGTGTTGCCCCAGAAGAG-3´ | 66 | 2360-2425 |
| qPCR\_Actin-rev | 5´-CACGATTAGCCTTGGGATTCA-3´ | | |
| qPCR\_OMT-for | 5´-ACCGGCGAGACTCAGATGAC-3´ | 265 | 10-274 |
| qPCR\_OMT-rev | 5´-AGGTTAGAATGGAGTAGCTGGC-3´ | | |
| qPCR\_CHS-for | 5´-GCTGTCAAGGCCATTAAGGA-3´ | 245 | 328-572 |
| qPCR\_CHS-rev | 5´-GAGCAAACAACGAGAACACG-3´ | | |
| GUSi-for | 5´-TTCGATGCGGTCACTCATTA-3´ | 414 | 5816-6230 |
| GUSi-rev | 5´-TAGAGCATTACGCTGCGATG-3´ | | |
| CHSi-for | 5´-GCTGTCAAGGCCATTAAGGA-3´ | 244 | 395-698 |
| CHSi-rev | 5´-GAGCAAACAACGAGAACACG-3´ | | |

## Slide 3
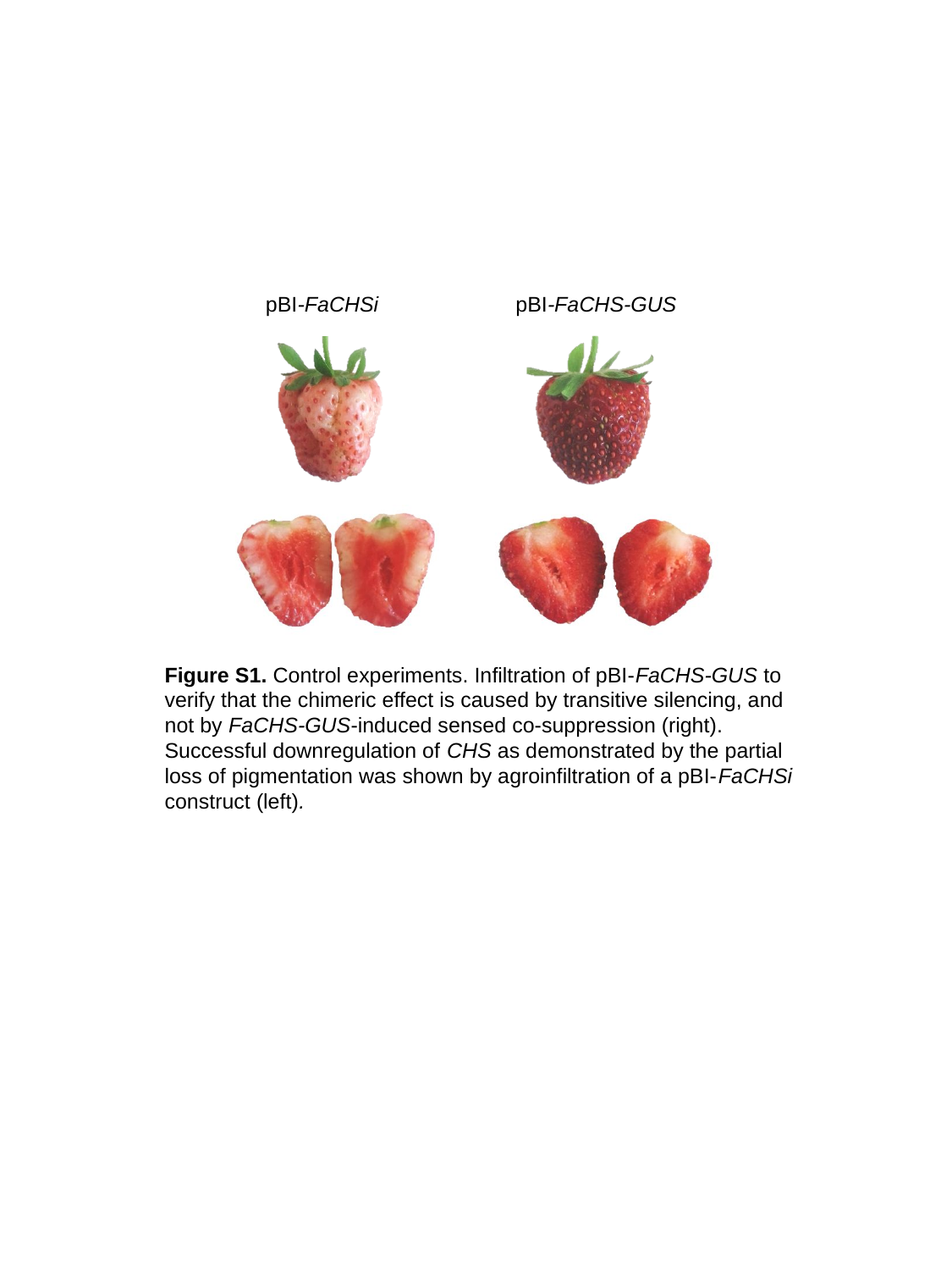

pBI-FaCHSi
pBI-FaCHS-GUS
Figure S1. Control experiments. Infiltration of pBI-FaCHS-GUS to verify that the chimeric effect is caused by transitive silencing, and not by FaCHS-GUS-induced sensed co-suppression (right). Successful downregulation of CHS as demonstrated by the partial loss of pigmentation was shown by agroinfiltration of a pBI-FaCHSi construct (left).

## Slide 4
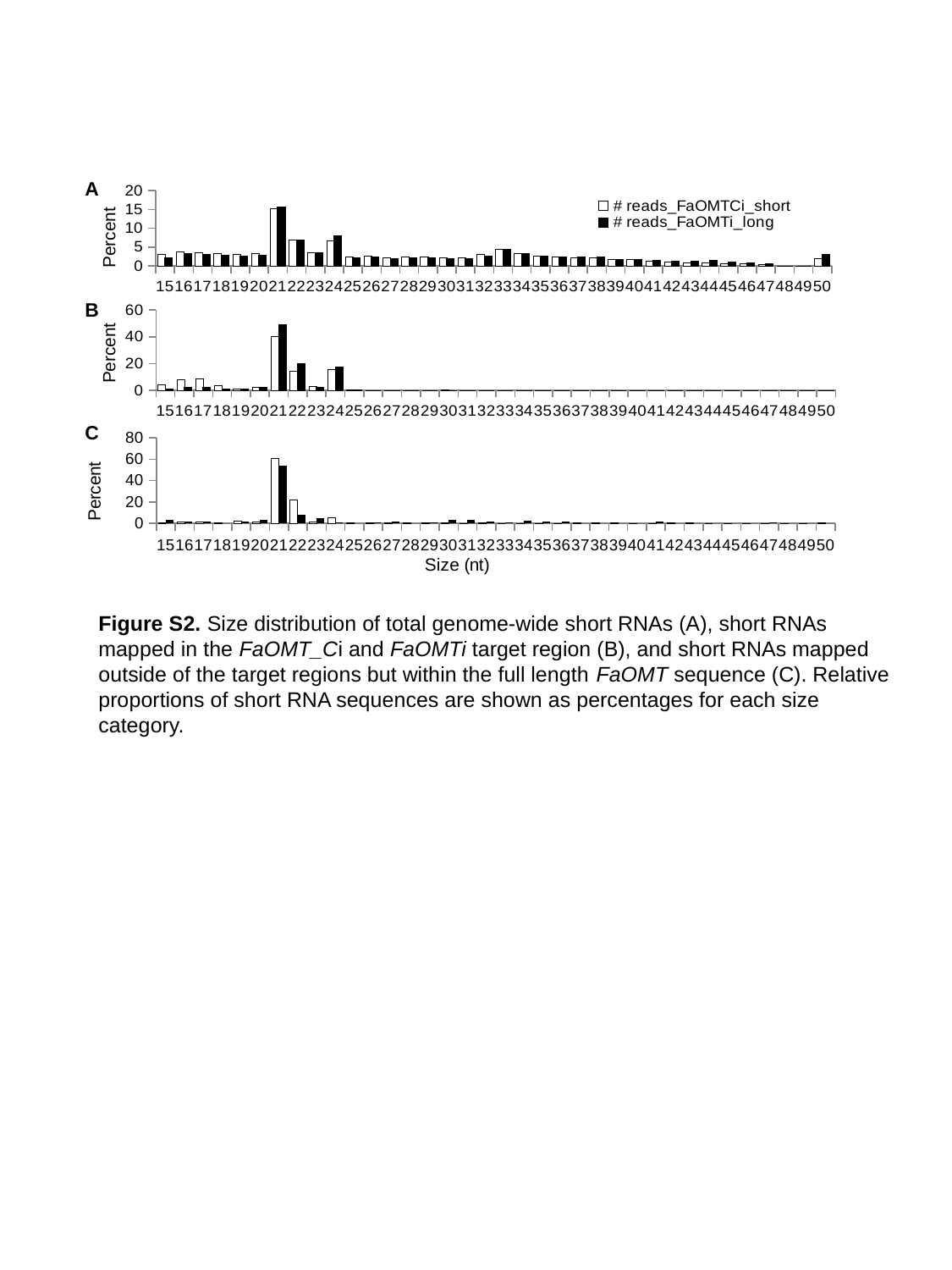

A
### Chart
| Category | # reads_FaOMTCi_short | # reads_FaOMTi_long |
|---|---|---|
| 15 | 2.97713782856824 | 2.152351318288137 |
| 16 | 3.8536680352403576 | 3.2058537629142756 |
| 17 | 3.459811540756079 | 3.0618613179665792 |
| 18 | 3.3891063533557797 | 2.876043917248054 |
| 19 | 3.1084164308490987 | 2.591356678164426 |
| 20 | 3.3666292306550507 | 2.868747305270922 |
| 21 | 15.1164597859398 | 15.655395788601483 |
| 22 | 6.870701980815833 | 6.780119283938254 |
| 23 | 3.529213406729638 | 3.4146129650946575 |
| 24 | 6.7060645563030965 | 8.039627914569357 |
| 25 | 2.330204241328581 | 2.078198939514288 |
| 26 | 2.7129411284022438 | 2.422176746473548 |
| 27 | 2.220787304408045 | 1.949217520433686 |
| 28 | 2.4022024063347547 | 2.1604238481033478 |
| 29 | 2.3691332110866172 | 2.064202575435441 |
| 30 | 2.1936502904157003 | 1.8569504444405187 |
| 31 | 2.238894162800879 | 1.969607745928092 |
| 32 | 2.974391544133341 | 2.5554480959088046 |
| 33 | 4.507413028507829 | 4.331188293548519 |
| 34 | 3.3338496623906173 | 3.244783948289261 |
| 35 | 2.6902209259116074 | 2.6595926834230577 |
| 36 | 2.462346552650178 | 2.4088070852638928 |
| 37 | 2.246662372257045 | 2.4737976649498736 |
| 38 | 2.0957770575605212 | 2.380284643966743 |
| 39 | 1.6208146638141856 | 1.8348144038104728 |
| 40 | 1.6357459692069658 | 1.813513967006034 |
| 41 | 1.3443139887505804 | 1.510025641846324 |
| 42 | 1.0814619852947072 | 1.3124650230787096 |
| 43 | 0.9123198268030638 | 1.2938579164621489 |
| 44 | 0.8727340244212438 | 1.5320870749919422 |
| 45 | 0.5814209979046001 | 1.027583804535554 |
| 46 | 0.5193735885533205 | 0.7497828362647013 |
| 47 | 0.4140062574944219 | 0.5562584824046842 |
| 48 | 0.0 | 0.0 |
| 49 | 0.0 | 0.0 |
| 50 | 1.8621256603559877 | 3.1689603618642055 |Percent
B
### Chart
| Category | # reads_FaOMTCi_short | # reads_FaOMTi_long |
|---|---|---|
| 15 | 3.849855630413846 | 1.2071463061323018 |
| 16 | 8.020532563362238 | 2.4142926122646067 |
| 17 | 8.341353865896691 | 2.4142926122646067 |
| 18 | 3.208213025344883 | 1.2071463061323018 |
| 19 | 1.12287455887071 | 1.0622887493964281 |
| 20 | 1.92492781520693 | 1.931434089811685 |
| 21 | 40.10266281681106 | 49.25156929019797 |
| 22 | 14.116137311517484 | 20.280057943022687 |
| 23 | 2.887391722810409 | 2.317720907774022 |
| 24 | 15.720243824189927 | 17.382906808305084 |
| 25 | 0.16041065126724421 | 0.28971511347175277 |
| 26 | 0.01604106512672442 | 0.009657170449058426 |
| 27 | 0.01604106512672442 | 0.009657170449058426 |
| 28 | 0.01604106512672442 | 0.009657170449058426 |
| 29 | 0.01604106512672442 | 0.009657170449058426 |
| 30 | 0.16041065126724421 | 0.009657170449058426 |
| 31 | 0.01604106512672442 | 0.009657170449058426 |
| 32 | 0.01604106512672442 | 0.009657170449058426 |
| 33 | 0.01604106512672442 | 0.009657170449058426 |
| 34 | 0.01604106512672442 | 0.009657170449058426 |
| 35 | 0.01604106512672442 | 0.009657170449058426 |
| 36 | 0.01604106512672442 | 0.009657170449058426 |
| 37 | 0.01604106512672442 | 0.009657170449058426 |
| 38 | 0.01604106512672442 | 0.009657170449058426 |
| 39 | 0.01604106512672442 | 0.009657170449058426 |
| 40 | 0.01604106512672442 | 0.009657170449058426 |
| 41 | 0.01604106512672442 | 0.009657170449058426 |
| 42 | 0.01604106512672442 | 0.009657170449058426 |
| 43 | 0.01604106512672442 | 0.009657170449058426 |
| 44 | 0.01604106512672442 | 0.009657170449058426 |
| 45 | 0.01604106512672442 | 0.009657170449058426 |
| 46 | 0.01604106512672442 | 0.009657170449058426 |
| 47 | 0.01604106512672442 | 0.009657170449058426 |
| 48 | 0.01604106512672442 | 0.009657170449058426 |
| 49 | 0.01604106512672442 | 0.009657170449058426 |
| 50 | 0.01604106512672442 | 0.009657170449058426 |Percent
C
### Chart
| Category | | |
|---|---|---|
| 15 | 0.3174603174603175 | 3.2786885245901636 |
| 16 | 1.26984126984127 | 1.6393442622950818 |
| 17 | 1.5873015873015872 | 1.6393442622950818 |
| 18 | 0.3174603174603175 | 0.0 |
| 19 | 2.2222222222222228 | 1.6393442622950818 |
| 20 | 0.9523809523809523 | 3.2786885245901636 |
| 21 | 60.63492063492063 | 54.09836065573771 |
| 22 | 21.58730158730159 | 8.196721311475407 |
| 23 | 0.9523809523809523 | 4.918032786885246 |
| 24 | 5.079365079365079 | 0.819672131147541 |
| 25 | 0.3174603174603175 | 0.0 |
| 26 | 0.3174603174603175 | 0.819672131147541 |
| 27 | 0.3174603174603175 | 1.6393442622950818 |
| 28 | 0.634920634920635 | 0.0 |
| 29 | 0.3174603174603175 | 0.819672131147541 |
| 30 | 0.3174603174603175 | 3.2786885245901636 |
| 31 | 0.0 | 3.2786885245901636 |
| 32 | 0.634920634920635 | 1.6393442622950818 |
| 33 | 0.0 | 0.819672131147541 |
| 34 | 0.0 | 2.4590163934426226 |
| 35 | 0.0 | 1.6393442622950818 |
| 36 | 0.0 | 1.6393442622950818 |
| 37 | 0.634920634920635 | 0.0 |
| 38 | 0.3174603174603175 | 0.0 |
| 39 | 0.3174603174603175 | 0.0 |
| 40 | 0.0 | 0.0 |
| 41 | 0.0 | 1.6393442622950818 |
| 42 | 0.3174603174603175 | 0.0 |
| 43 | 0.3174603174603175 | 0.0 |
| 44 | 0.0 | 0.0 |
| 45 | 0.0 | 0.0 |
| 46 | 0.0 | 0.0 |
| 47 | 0.0 | 0.819672131147541 |
| 48 | 0.0 | 0.0 |
| 49 | 0.0 | 0.0 |
| 50 | 0.3174603174603175 | 0.0 |Figure S2. Size distribution of total genome-wide short RNAs (A), short RNAs mapped in the FaOMT_Ci and FaOMTi target region (B), and short RNAs mapped outside of the target regions but within the full length FaOMT sequence (C). Relative proportions of short RNA sequences are shown as percentages for each size category.
